# EVX2 methylation assay enables risk assessment of cervical cancer in high-risk HPV-infected individuals

**DOI:** 10.1186/s12885-026-16256-z

**Published:** 2026-06-08

**Authors:** Jingyi Wu, Wenyuan He, Weiyun Zhang, Danli Ye, Wen zhi Cui, Yuansen Li, Min Luo, Wei Wang

**Affiliations:** 1 Graduate School, Guangzhou University of Chinese Medicine, Guangzhou, 510006 China; 2 Department of Pathology, General Hospital of Southern Theater Command, People’s Liberation Army of China, 111Liuhua Road, Guangzhou, Guangdong 510010 China; 3 Department of Laboratory Medicine, General Hospital of Southern Theater Command, People’s Liberation Army of China, Guangzhou, 510010 China; 4 Department of Obstetrics and Gynecology, General Hospital of Southern Theater Command, People’s Liberation Army of China, 111 Liuhua Road Guangzhou, Guangzhou, 510010 China

**Keywords:** Cervical cancer, EVX2, Human papillomavirus, Screening, Methylation

## Abstract

**Background:**

Stratifying cancer risk in high-risk human papillomavirus (hr-HPV) infected individuals can effectively reduce colposcopy referrals and minimize invasive procedures. Current assay methods need to be improved and perfected.

**Methods:**

Four methylation markers (ZNF135, EVX2, MARCHF11, and UBD) were identified to discriminate cervical cancer (CC) from normal mucosal tissues through the analysis of TCGA/GEO cohorts. Subsequently, a specific methylation marker (EVX2) was further identified via immunohistochemical (IHC) assays and an extensive literature review. After validating the applicability of this technique for EVX2 methylation detection via MALDI-TOF mass spectrometry, a methylation model was constructed based on the selected markers in a retrospective cohort of cervical exfoliated cells (*n* = 104) and validated in an independent cohort (*n* = 22) using the identical technique and random forest algorithm. The diagnostic performance was systematically compared with LCT and high-oncogenicity HPV16/18 testing.

**Results:**

Four methylation markers (ZNF135, EVX2, MARCHF11, and UBD) were identified that could discriminate CC from normal squamous epithelia. Among these markers, EVX2 was further pinpointed as the key candidate based on gene expression and literature review. A diagnostic model constructed via EVX2 methylation status exhibited robust diagnostic performance in differentiating patients with squamous intraepithelial lesion (SIL) or CC from healthy normal controls, and its diagnostic efficacy was significantly superior to that of LCT and HPV16/18 assays (AUC: 0.95 vs. 0.85 for LCT and 0.57 for HPV16/18 testing). This superiority was more pronounced in detecting HSIL and CC, with a detection rate of 96.97% versus 57.14% (LCT) and 37.5% (HPV16/18 testing). Subsequent analyses of TCGA databases further validated the diagnostic value of EVX2 methylation in CC.

**Conclusions:**

EVX2 methylation detection via MALDI-TOF mass spectrometry reliably discriminates normal squamous epithelium from HSIL and above cervical malignant lesions, and may serve as a rapid, non-invasive, and promising tool for cancer-risk screening and triage in hr-HPV-positive patients.

**Supplementary Information:**

The online version contains supplementary material available at 10.1186/s12885-026-16256-z.

## Introduction

Cervical cancer (CC) is one of most common causes of cancer-related death among women worldwide [1]. Detection of hr-HPV types is a standard method for CC screening [2]. Although its higher sensitivity, hr-HPV testing has several limitations, including low specificity, high cost and a high misdiagnosis rate of cervical adenocarcinoma [3]. Notably, most women testing positive for HPV16 and HPV18 are routinely referred for immediate colposcopy, a practice that may trigger substantial patient anxiety and lead to overtreatment [4, 5].

Increased methylation of tumor suppressor genes is an early event in many tumors, and changes in DNA methylation patterns are closely associated with the tumorigenesis of CC [6, 7]. Specifically, hr-HPV infection has been demonstrated to induce promoter hypermethylation of multiple tumor suppressor genes, including APC, SFRP1, PTEN, and PAX1, by upregulating DNA methyltransferases (DNMTs) and interacting with histone modifiers such as EZH2 [8–10]. However, although several methylation markers have been incorporated into CC screening protocols, most of these assays depend on multigene panels that incur substantial clinical costs, and the specificity of single-gene methylation assays remains suboptimal [11–13]. Therefore, there is an urgent need to develop a more efficient and cost-effective non-invasive screening approach to stratify and triage the risk of carcinogenesis in high-risk HPV-infected individuals, thereby reducing the referral rate of invasive procedures such as colposcopy directed biopsy collection. We aim to perform an exploratory study that first identifies methylation biomarkers via database mining, and further refines the biomarker panel through literature review and functional validation. A diagnostic model was constructed and validated based on the finally identified biomarkers which was designed to assess their performance as non-invasive biomarkers for stratifying oncogenic risk in hr-HPV-positive women.

## Materials and methods

### Database acquisition and bioinformatic analysis

Differential methylation loci (DMLs) were identified from 102 squamous cell carcinoma (SCC) tissues and 29 normal cervical epithelium samples obtained from the Gene Expression Omnibus (GEO) dataset (GSE211668) via a moderate t-test, with cut-off criteria set at *P* ≤ 0.01 and a mean difference>0.35. Similarly, differential methylation analysis was performed on methylation data derived from 306 CC samples in The Cancer Genome Atlas (TCGA) and 656 healthy leukocyte samples from GSE40279. Subsequently, the intersection of DMLs identified from these two groups was extracted. Pearson correlation coefficients (r2) between β values of any two closely positioned CpGs (within 200 bp) were calculated, and a cut-off of r2>0.5 was used to identify the methylation-correlated blocks (MCBs). Least absolute shrinkage and selection operator (LASSO) were used for variable selection. Statistical analyses (including t-test and Wilcoxon) were performed using R (version 3.6.0) and SPSS 25.0 (IBM SPSS Statistics 25, IBM, Somers, IL, USA). Heatmaps were created using the “Complex Heatmap” package (version 2.14.0).

### Immunohistochemical (IHC) detection of the formalin fixed paraffin embedded (FFPE) samples

We identified the critical tumor suppressor genes in CC from the genes selected from databases through a comprehensive literature review and further confirmed via IHC assay. The rabbit anti-human polyclonal antibodies EVX2 (ThermoFisher, PA5-59632) and ZNF135 (Sigma, HPA006961) were finally diluted at 1:150. The immunohistochemical staining was performed on the FFPE specimens from 58 normal cervical epithelium, 30 HSIL and 55 CC following the manufacturers’ instructions. A total of 143 FFPE specimens were collected from General Hospital of Southern Theater Command. Negative control sections were incubated with PBS instead of primary antibody. The sections were independently examined by two experienced pathologists over 10 years of experience, who were blinded to the clinicopathological information. Both EVX2 and ZNF135 immunohistochemical staining was localized to the nucleus. The staining intensity was quantified as follows: positive cells with <10% staining were scored as negative staining (-, 0); cells with 10–49% staining were scored as (+, 1); cells with 50–74% staining were scored as (++, 2); and cells with 75–100% staining were scored as (+++, 3) [14].

### Cervical exfoliated cell sample collection, DNA extraction and HPV-DNA genotyping

A total of 3917 cervical exfoliated cells were collected with a specialized brush from participants at the General Hospital of the Southern Theater Command between May 2023 and April 2024, then stored in DNA-preserving buffer at 4 °C. Genomic DNA was extracted from these cells using the DNA Extraction Kit (Tellgen, Jiangxi, China). One aliquot was used for HPV-DNA genotyping, and another was bisulfite-converted with the EZ-96 DNA Methylation Gold Kit (TANTICA, Nanjing, China) according to the manufacturer’s instructions. Following bisulfite treatment, methylated cytosines (C) within CpG sites exhibited remarkable stability and remained unaltered, while unmethylated cytosines (C) were converted into uracil (U). HPV DNA genotyping was performed using the HPV 27 Genotyping Assay (Tellgen, Shanghai, China), which simultaneously detects 27 human papillomavirus types: 17 high-risk types (16, 18, 31, 33, 35, 39, 45, 51, 52, 56, 58, 59, 66, 68, 26, 53, 82) and 10 low-risk types (6, 11, 40, 42, 43, 44, 55, 61, 81, 83). After HPV genotyping, the residual DNA samples were stored at − 20 °C for subsequent methylation detection.

### Cell and tissue sample collection and screening

A total of 467 cervical exfoliated cell samples were screened for hr-HPV infection. Among them, 104 pathologically confirmed cases by liquid-based cytology (LCT) and/or colposcopic biopsy were used to establish a risk prediction model. Another independent set of 22 h-HPV positive samples was collected from gynecology clinic patients with confirmed persistent hr-HPV infection prior to colposcopically directed biopsy. Exclusion criteria included HPV-related anogenital lesions, HPV vaccination, pregnancy, non-cervical malignancies, and immunocompromised status. The clinical characteristics of all 126 enrolled cases are summarized in Table 1, which includes 6 cases of cervical squamous cell carcinoma (SCC), 3 cases of endocervical adenocarcinoma (ECA), 23 cases of high-grade squamous intraepithelial lesion (HSIL), 26 cases of low-grade squamous intraepithelial lesion (LSIL), and 68 cases of normal cervical epithelium. The mean age was 45 years, ranging from 22 to 73 years. Furthermore, 5 normal and 15 CC FFPE tissue samples previously used for IHC were randomly selected for DNA extraction and subsequent EVX2 methylation analysis to detect aberrant methylation. This retrospective study utilized archived clinical samples and data collected between May 2023 and April 2024, and the protocol was approved by the Ethics Committee of the General Hospital of Southern Theater Command (No. NZLLKZ2025096).

**Table 1:** Clinical characteristics of the 126 qualified cervical exfoliated cell cohort

Characteristics	Diagnosis				
	Normal	LSIL	HSIL	CC	Total
Total (n)	68	26	23	9*	126
Age (years)	44(22 ~ 69)	46(29 ~ 64)	42(27 ~ 62)	59(33–73)	45(22–73)
≤ 40	26(38%)	8(31%)	10(43%)	1(11%)	45(36%)
>40	42(62%)	18(69%)	13(57%)	8(89%)	81(64%)
Hr-HPV subtype					
HPV16/18	19(28%)	6(23%)	8(35%)	4(44%)	37(29%)
Other	49(72%)	20(77%)	15(65%)	5(56%)	89(71%)
LCT					
Positive	4(6%)	21(81%)	11(48%)	4(45%)	40(32%)
Negative	64(94%)	5(19%)	11(48%)	2(22%)	82(65%)
NA	0	0	1(4%)	3(33%)	4(3%)
Group					
Training cohort	39(54%)	19(26%)	9(13%)	5(7%)	72
Validation cohort	17(53%)	7(22%)	8(25%)	0	32
Additional validation	12(55%)	0	6(27%)	4(18%)	22

* including 3 cases of cervical adenocarcinoma (ECA)

### Methylation detection based on matrix-assisted laser desorption ionization time-of-flight (MALDI-TOF) mass spectrometry

All 20 formalin-fixed paraffin-embedded (FFPE) tissue specimens and 126 cervical exfoliated cell samples were subjected to methylation detection using MALDI-TOF mass spectrometry. The bisulfite-specific primers were designed and synthesized by Sangon (Sangon Biotech, Shanghai, China). We designed a 192 bp amplicon located in the promoter region of the EVX2 gene, which covered seven CpG sites contained in the EVX2 amplicon176948693-176978459. No known single-nucleotide polymorphisms were identified within the primer-binding regions or at CpG sites. Target sequences were PCR-amplified, and quantitative DNA-methylation levels were determined by MALDI-TOF mass spectrometry (Agena Bioscience, San Diego, CA, USA) according to the protocol detailed by Lei et al. [15]. Briefly, amplicons were dephosphorylated with shrimp alkaline phosphatase (SAP) and subjected to in-vitro transcription with T7 polymerase plus RNase A digestion following the Agena EpiTYPER protocol (Product ID: 10667). After resin cleanup, the cleavage products were dispensed onto a SpectroCHIP array via the MassARRAY Nanodispenser. The chip was read on the MassARRAY analyzer. Data were acquired with SpectroACQUIRE v3.3.1.3 and analyzed with EpiTYPER v1.3.

### Development and validation of the methylation screening model

Methylation data were generated using the Agena MassARRAY EpiTYPER system. Raw signals were processed to obtain methylation β-values for each CpG locus within EVX2, followed by quality control to exclude low-quality values. A risk predictive model was constructed using the randomForest package in R, with normalized EVX2 β-values as input features for model training and validation. Diagnostic performance was evaluated by receiver operating characteristic (ROC) analysis using the pROC package. Heatmaps were generated with the ComplexHeatmap package (version 2.14.0). All statistical tests were two-sided, and *P* < 0.05 was considered statistically significant.

## Results

### Discovery of DNA methylation markers to distinguish cervical cancer from normal tissues based on public databases

**Fig. 1:**
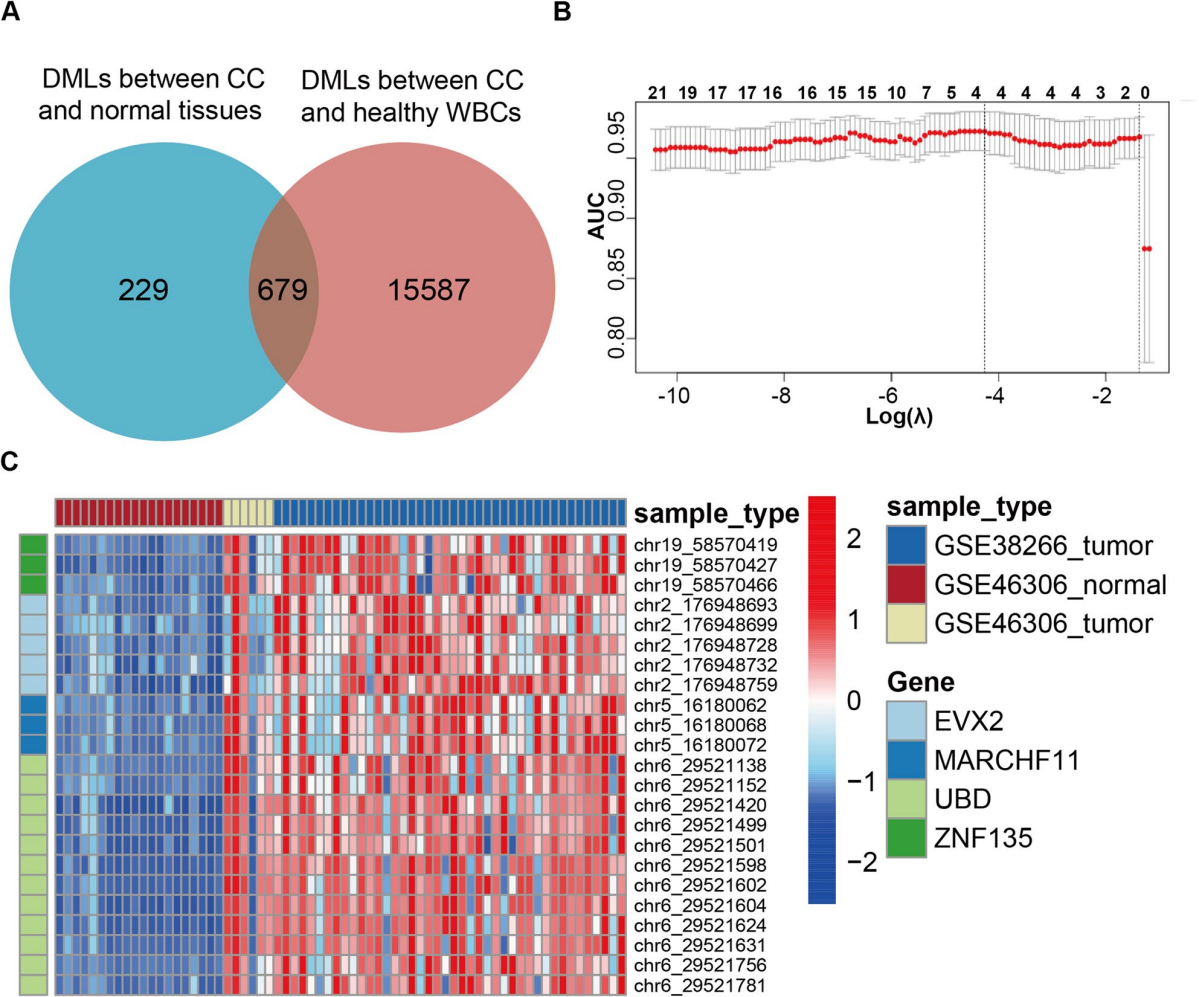
Screening of DNA methylation markers to distinguish cervical cancer from normal tissue. **A** A total of 679 common DMLs between normal tissues and CC were identified after excluding the interference of leukocyte DNA. **B** Four methylation markers were identified by LASSO for the optimal discrimination of CC from normal epithelium. **C** GSE dataset confirmed four methylation markers can effectively distinguish CC from normal tissues. The red colour represents a high methylation level, whereas the blue colour indicates a low methylation level

Differential methylation analysis was performed on 102 CC and 29 normal tissues from the GEO dataset. To eliminate the influence of leukocyte DNA in cervical mucus, we further analyzed DNA methylation profiles of 306 CC tissues from TCGA and 656 normal blood samples from a dataset (GSE40279). We selected the commonly shared 679 differential methylation loci (DMLs) by intersecting the above two sets which were finally assembled into 31 MCBs using the Pearson correlation method with an r^2^ cut-off of 0.5 (Fig. 1A). LASSO regression was applied for variable selection. Finally, four methylation markers (ZNF135, EVX2, MARCHF11, and UBD) were identified to optimally distinguish CC from normal squamous epithelium, with the AUC reaching its maximum value of 0.955 (Fig. 1B). GSE dataset displayed high and stable methylation in tumors, but very low levels in normal tissues which suggest the screened methylation markers could be used to diagnose CC.

### Further screening of methylation biomarkers and its functional validation in cervical cancer

**Fig. 2:**
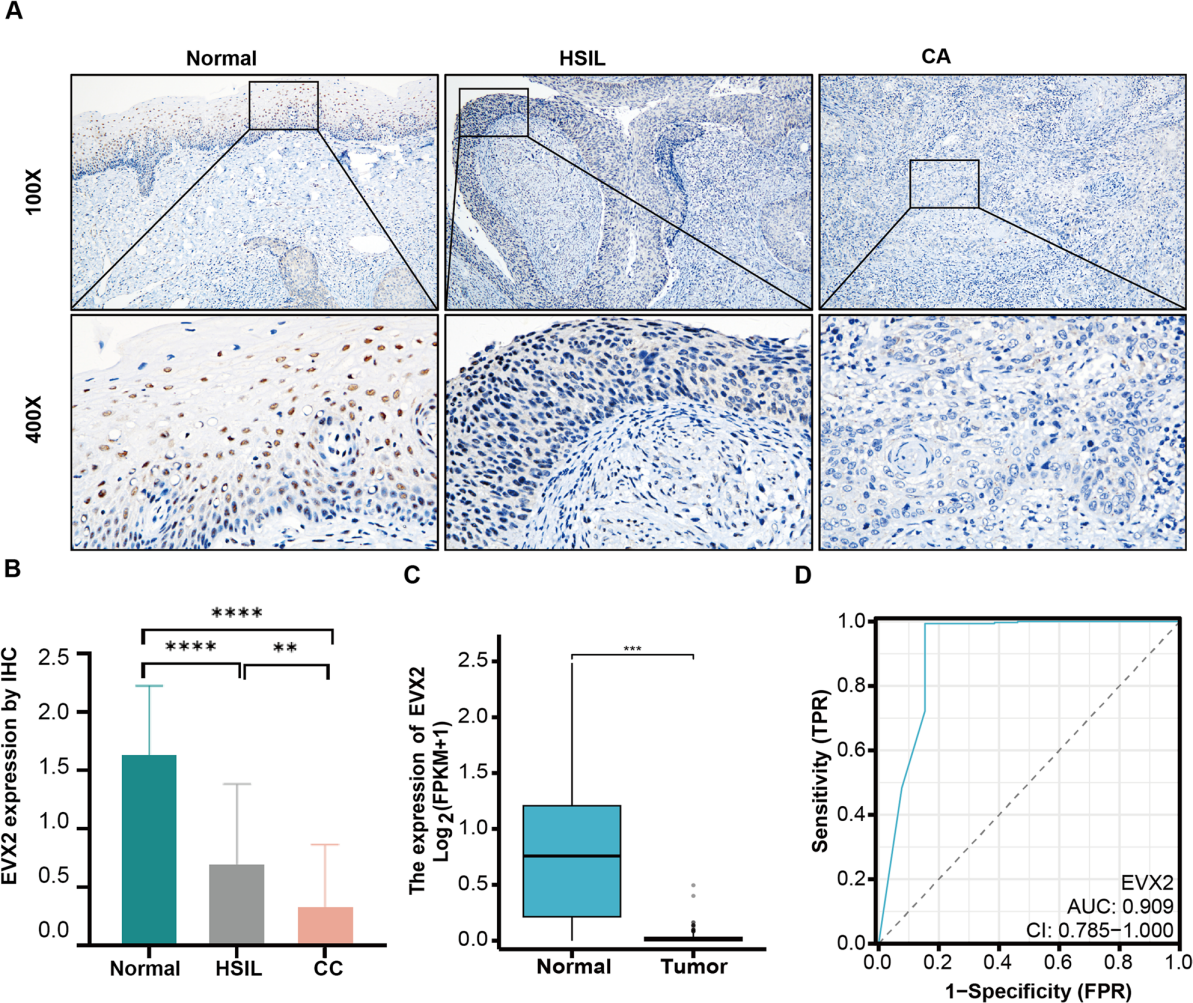
Expression and biological significance of EVX2 in normal tissues, HSIL and cervical cancer. **A** IHC demonstrated high EVX2 expression in normal epithelium, with marked reduction in HSIL and CC. **B** Quantitative analysis indicated significantly higher EVX2 expression in normal tissues compared to HSIL and CC. **C** Analysis of TCGA data confirmed significantly elevated EVX2 expression in normal tissues relative to CC. (D)ROC curve analysis showed robust performance of EVX2 in discriminating tumor tissues from normal tissues

A comprehensive literature review indicates that the promoter regions of EVX2 and ZNF135 are frequently hypermethylated in cancer, endowing them with the potential to serve as diagnostic and prognostic biomarkers for tumors [16–20]. IHC was conducted to evaluate the protein expression levels of EVX2 and ZNF135 in FFPE tissue samples. The results revealed that EVX2 protein was highly expressed in normal epithelium (55 out of 58 samples), but its expression was significantly reduced in HSIL samples (18 out of 30) and CC samples (17 out of 55). Quantitative analysis demonstrated that the expression of EVX2 in normal tissues was significantly higher than that in HSIL and CC (*P* < 0.001, Fig. 2B, Table S1). Moreover, EVX2 expression in HSIL was higher than in cervical cancer (*P* < 0.01). Additionally, the expression of EVX2 was not significantly associated with age (<50 years vs. ≥50 years), tumor differentiation (high-middle vs. low), clinical stage (stage I/II vs. stage III), tumor size (< 2 cm vs. ≥2 cm), high-risk human papillomavirus (hr-HPV) type (HPV16/18 vs. other types), or invasion depth (< 1/2 vs. ≥1/2) (Table S2). However, ZNF135 was highly expressed in both normal epithelium and HSIL, as well as in CC, with no significant differences in expression levels (*P* > 0.05).

Further analysis using data from TCGA confirmed that EVX2 expression was significantly lower in CC tissues compared to normal tissues, suggesting its potential role as a tumor suppressor gene (Fig. 2C). The ROC curve analysis revealed that EVX2 has an extremely strong ability to distinguish between tumor and normal tissues, with an AUC value of 0.909 (Fig. 2D). The aforementioned findings suggest that EVX2 may play a potential role in the progression of normal epithelium to precancerous and cancerous lesions, and could serve as a promising diagnostic biomarker. Accordingly, the present study aimed to explore the utility of EVX2 methylation detection in cervical exfoliated cells for assessing the carcinogenic risk in patients infected with hr-HPV.

### Identification and validation of EVX2 methylation in cervical HSIL and carcinoma

**Fig. 3:**
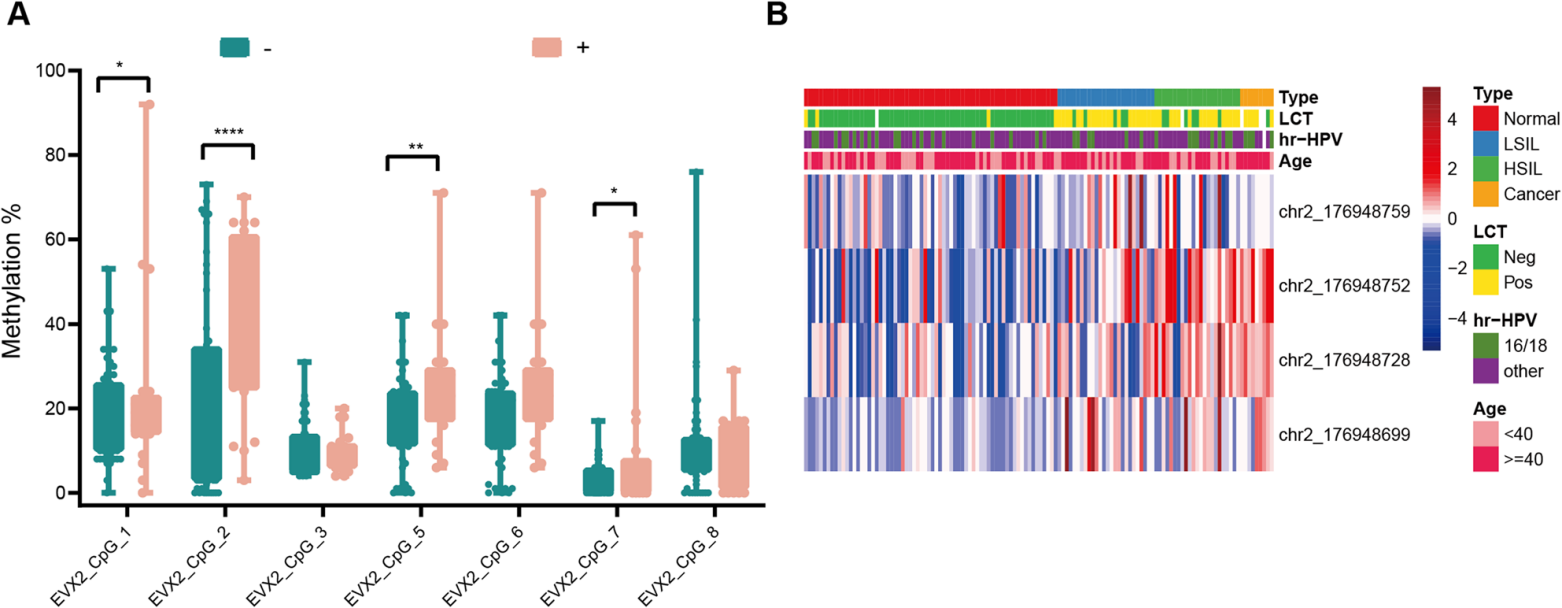
Identification and validation of differential EVX2 CpG site. **A** Four significantly differentially methylated EVX2 CpG site were identified. **B** A clustering heatmap of 104 high-risk HPV-infected individuals demonstrated significantly higher methylation levels at four selected EVX2 CpG sites in LSIL, HSIL, and CC groups, compared to the normal group

MALDI-TOF mass spectrometry was initially performed in FFPE samples from 5 normal epithelia and 15 CC samples to detect the methylation levels of 7 sites of EVX2 gene, namely EVX2_CpG_1, EVX2_CpG_2, EVX2_CpG_3, EVX2_CpG_5, EVX2_CpG_6, EVX2_CpG_7, and EVX2_CpG_8. A moderate t-test was performed to compare the EVX2 methylation levels and the methylation status at each CpG locus between normal epithelia and CC. The results demonstrated that EVX2 exhibited significantly higher methylation in CC relative to normal controls, with the EVX2_CpG_1, EVX2_CpG_2, EVX2_CpG_5 and EVX2_CpG_7 loci showing the most prominent hypermethylation (*p* < 0.05). These four loci were thus selected to construct a random forest-based diagnostic model for non-invasive cancer risk stratification in hr-HPV-infected individuals (Fig. 3A). Consistently, a clustering heatmap of 104 h-HPV-infected individuals revealed significantly higher EVX2 methylation levels in the HSIL and CC groups than in the normal and LSIL groups (Fig. 3B), although no significant differences in methylation levels at these loci were found between HPV16/18 infections and other high-risk HPV infections (Supplementary Fig. S1). The results suggested that EVX2 methylation detection may be feasible for cancer risk stratification in hr-HPV infected individuals.

### Construction and validation of a non-invasive diagnostic model for cancer risk stratification in hr-HPV infected individuals by using EVX2 methylation

**Fig. 4:**
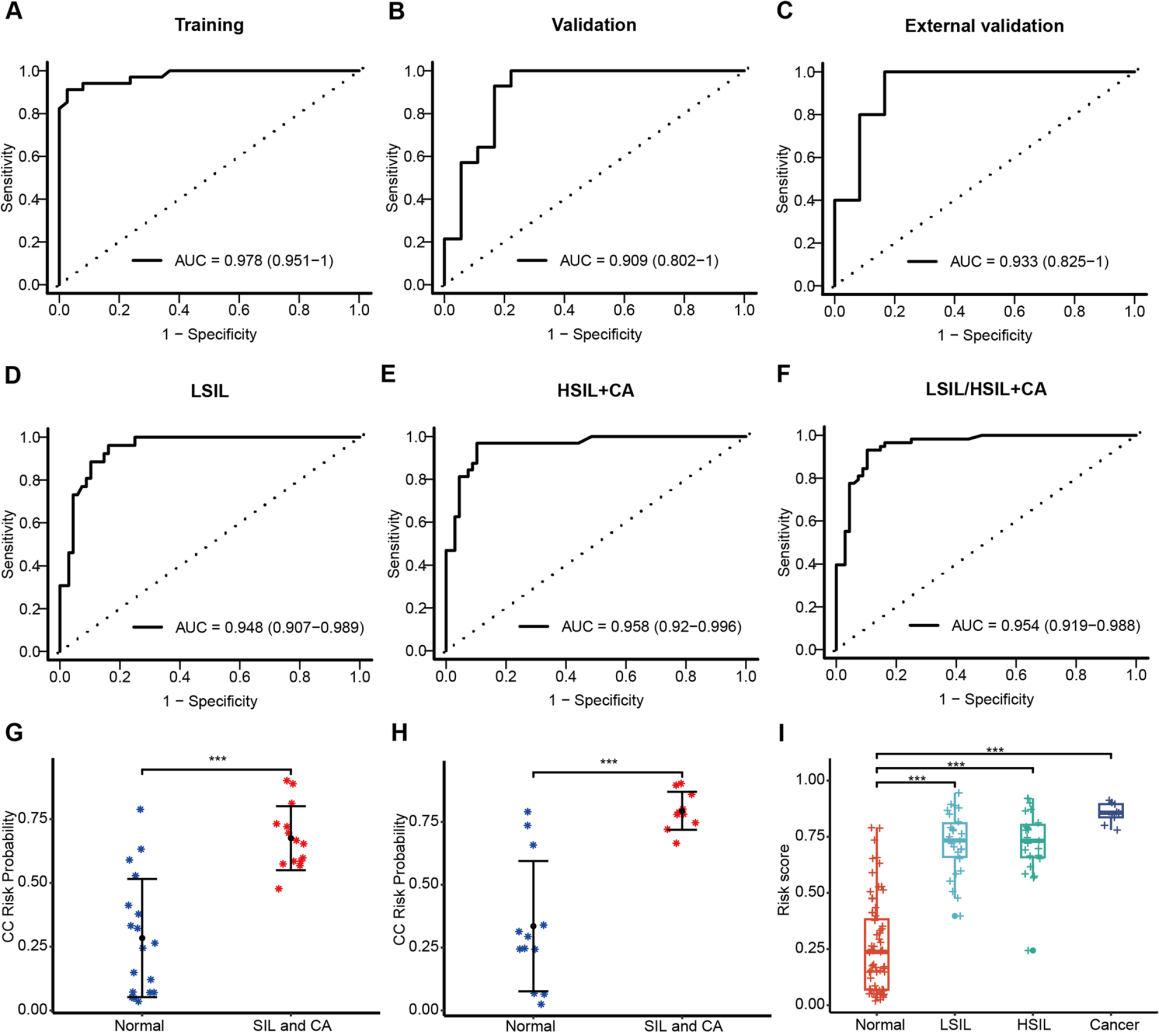
The performance and risk score of EVX2 methylation model in the non-invasive risk stratification in hr-HPV infected individuals. **A-C **AUC values for EVX2 methylation were 0.948 (0.951–1), 0.909 (0.802–1), and 0.933 (0.825–1) in the training, validation, and additional cohorts, respectively. **D-F** For discriminating LSIL, HSIL, and CC from healthy controls, EVX2 yielded AUCs of 0.948, 0.958, and 0.954, respectively. **G**,**H** Notably, risk scores were significantly higher in SIL or CC patients than in healthy controls across validation and additional cohorts. **I **The risk score of the model in healthy controls (Normal) and in patients with LSIL, HSIL and CC. ***P < 0.001

Cervical exfoliated cell samples from 104 h-HPV infected individuals were randomly assigned to the training cohort and validation cohort at a ratio of 2:1. Following the aforementioned CpG sites screening, a methylation prediction model was constructed based on the four selected EVX2 methylation sites using the random forest algorithm, which generates a unique probability value for each individual sample. By inputting the methylation levels of these four EVX2 sites into the model, a final risk score is generated, which enables the stratification of hr-HPV-positive patients into risk categories for the development of SIL or CC. The ROC curves demonstrated the EVX2 methylation model achieved AUC values of 0.978 and 0.909 in the training and validation cohorts, respectively (Fig. 4A, B), with sensitivities of 96.67% and 93.33%, and specificities of 92.87% and 82.35% in distinguishing normal tissues from cervical precancerous lesions and CC in these two cohorts (Table S3A, B). Furthermore, in an additional validation set containing 22 h-HPV-infected individuals, the AUC curve for distinguishing CC patients from normal controls also reached 0.993, with a sensitivity of 90% (Fig. 4C, Table S3C). We then evaluated the performance of EVX2 methylation in separating LSIL, HSIL and CC patients, as well as all cervical lesions from healthy controls, and found that the AUC was 0.948, 0.958 and 0.954, respectively (Fig. 4D-F). Furthermore, the risk score that was calculated based on the EVX2 methylation was consistently lower in healthy controls (Normal) compared to patients with squamous intraepithelial lesions (SIL) and CC in both the validation and additional cohorts (Fig. 4G, H). Specifically, the risk scores were significantly higher in patients with LSIL, HSIL and CC compared to normal controls (Fig. 4I). These data indicated that EVX2 methylation model could serve as a robust and non-invasive method for cancer risk evaluation of hr-HPV infected patients.

### Comparative evaluation of EVX2 methylation, LCT and HPV16/18 detection for identifying HSIL and CC

**Fig. 5:**
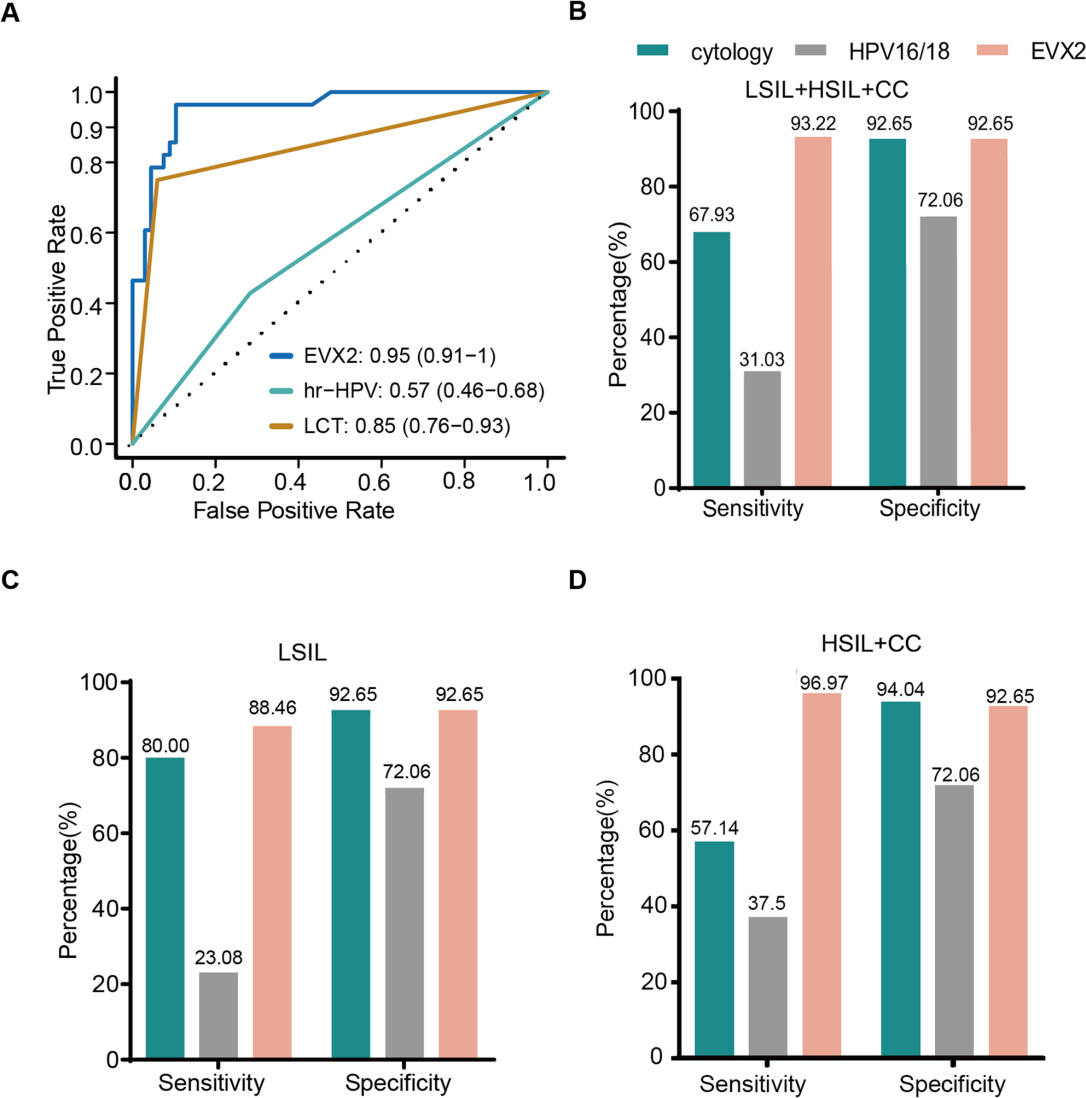
Comparison of the diagnostic performance in detecting SIL and CC between the EVX2 methylation and LCT and HPV16/18. **A** EVX2 methylation demonstrated superior diagnostic performance for SIL and CC, compared to LCT and HPV16/18. EVX2 methylation exhibited higher sensitivity and specificity than LCT and HPV16/18 detection in the detection of all cervical lesions (**B**), LSIL (**C**), and HSIL/CC (**D**)

Currently, LCT and high-risk HPV 16/18 detection are among the most commonly used CC screening methods. To assess the performance of EVX2 methylation as a CC screening tool, we conducted a comparative analysis between EVX2 methylation, LCT, and hr-HPV 16/18 monitoring. A comparative analysis was conducted to assess the performance of EVX2 methylation as a screening tool for CC, in comparison to LCT and monitoring of high-risk HPV 16/18. Overall, EVX2 methylation was shown to be superior to both LCT and hr-HPV 16/18 in CC screening, with an AUC of 0.95 versus 0.57 and 0.85, respectively (Fig. 5A). Additionally, integrated analysis of 126 cases in this study revealed that EVX2 methylation achieved an overall sensitivity of 93.22% and specificity of 92.65% (Fig. 5B). Notably, in patients with LSIL, the sensitivity of EVX2 was higher compared with LCT and HPV16/18 (88.4% vs. 80% vs. 23.08%, Fig. 5C). Moreover, in the detection of HSIL and CC patients, EVX2 methylation exhibits markedly enhanced sensitivity, with a sensitivity of 96.97% compared to 57.14% for LCT and 37.5% for HPV16/18. Collectively, EVX2 methylation exhibited markedly enhanced sensitivity (96.97%), compared with 57.14% for LCT and 37.5% for hr-HPV 16/18. Collectively, these findings indicate that EVX2 methylation exhibits significantly improved sensitivity compared with LCT and high-oncogenic-potential hr-HPV 16/18, particularly in patients with high-grade lesions and above.

## Discussion

Cervical cancer ranks as the fourth most common malignancy among women [21], causing the death of 340,000 women globally each year. Persistent high-risk HPV infections are the leading cause of HSIL and CC. Cervical cytology and/or hr-HPV screening has significantly reduced the incidence and mortality of CC [22], but these methods also have certain limitations. Cytology screening is easily affected by human factors such as slide preparation and result interpretation. hr-HPV DNA testing increased the sensitivity of screening. However, most transient HPV infections test positive, which not only aggravates patients’anxiety but also markedly increases physical trauma from colposcopy. Therefore, an accurate, robust, and non-invasive test is highly desirable for cancer risk stratification in high-risk HPV infected individuals.

Altered DNA methylation patterns have been proven to be a promising strategy for cancer screening [23–25]. Multiple studies have shown that host and HPV methylation events are commonly seen in the early stages of cervical cancer development [26–28]. Methylation of host gene promoter sequences takes place in a wide range of genes including tumor suppressor genes, histone modification genes, and microRNAs (miRNA) but also in HPV viral genes [29, 30]. Numerous studies have shown that methylation has favorable screening sensitivity for cervical intraepithelial neoplasia (ClN2) or more severe lesions (ClN2+, or HSIL) as a triage method for women with positive hr-HPV status [31–34]. Cheng et al. performed combined methylation detection of three genes (FAM19A4, EPB41L3, and PAX1), which exhibited a sensitivity of 88.0% and a high specificity of 97.7% for the diagnosis of HSIL and CC, and the diagnostic sensitivity decreased when a single methylation marker was used individually [35]. The hypermethylation of the FAM19A4 and miR124-2 promoter regions has been confirmed as a key epigenetic event in the progression of cervical squamous cell carcinoma, with a sensitivity of 97.6% and specificity of 85.7% for diagnosing HSIL and above (HSIL+) [36]. Collectively, these findings confirmed that methylation detection possesses distinct advantages over traditional HPV testing in enhancing screening specificity. Given that most previous studies have focused on multigene methylation assays, the present study was designed to identify the minimum number of markers necessary for effective CC screening. Following database mining, comprehensive literature review, and immunohistochemical validation, the EVX2 gene was ultimately identified as the target marker.

EVX2 (Even-Skipped Homeobox 2) locates at the 5’ end of the chromosome 2 HOXD gene cluster with close correlation to the cluster, and its methylation has emerged as a clinically relevant epigenetic biomarker in multiple malignancies. Hypermethylation of EVX2 is observed in both lung squamous cell carcinoma and adenocarcinoma, implicating it as a tumor suppressor in lung tumorigenesis [18, 19]. Additionally, EVX2 methylation predicts platinum-based chemotherapy resistance in ovarian cancer [20] and enables prognostic stratification and individualized treatment decision-making in gastric cancer [37]. Collectively, these findings confirm EVX2 methylation as a promising biomarker for cancer early screening, therapeutic response assessment and prognostic prediction. Given this well-established potential, the clinical application of EVX2 methylation largely depends on appropriate detection methods. Existing techniques (e.g., qMSP, genome bisulfite sequencing) vary in sensitivity, efficiency, cost, and suitability for large-scale screening, limiting their clinical utility. In contrast, MALDI-TOF-MS offers distinct advantages: high-resolution, high-throughput quantification of multiple CpG sites, even in low-methylation samples, and sensitive single-CpG detection via single nucleotide amplification without normalization [33]. Thus, we used MALDI-TOF-MS to detect EVX2 methylation in cervical exfoliated cells, exploring its ability to stratify carcinogenic risk in HR-HPV-infected individuals.

To determine whether EVX2 gene methylation can be used for risk stratification of malignant transformation in individuals with high-risk HPV infection. We first confirmed the ability of EVX2 methylation in distinguishing CC from normal cervical tissues. We then constructed an EVX2 methylation-based diagnostic model with high performance in differentiating precancerous lesions, CC, and normal individuals. Specifically, its sensitivity for detecting precancerous lesions and CC was 91% in the validation cohort and 90% in the surveillance cohort, indicating good model stability and reproducibility. Notably, EVX2 methylation outperformed in distinguishing HSIL, CC from normal individuals compared to LSIL, with 96.67% sensitivity and 92.65% specificity. This is clinically significant because HSIL and CC are often missed by LCT, highlighting the need for more reliable screening methods. Accurate identification of HSIL and CC is critical for early intervention to reduce CC incidence and mortality.

Compared to conventional screening techniques, such as LCT and testing for high-risk HPV16/18, EVX2 methylation demonstrated substantially improved performance in detecting all precancerous lesions and CC, with a sensitivity of 93.62% versus 67.93% and 31.03%, and a specificity of 92.65% versus 72.06% and 92.65%. Notably, EVX2 methylation showed even greater superiority in diagnosing HSIL and CC specifically, achieving a sensitivity of 96.67% compared to 57.14% and 37.5%, and a specificity of 94.04% compared to 72.06% and 92.65%. In addition, cervical glandular lesions pose a significant challenge in cytology-based screening due to their high rates of missed diagnosis. Notably, all three cases of cervical adenocarcinoma in our cohort were correctly identified by the EVX2 methylation assay. If validated in subsequent large-scale studies, EVX2 methylation could serve as a promising biomarker for improving the diagnosis of glandular abnormalities. These results make EVX2 methylation detection based on MALDI-TOF-MS attractive for use in clinical decision making across a variety of patients and situations.

However, several limitations should be noted. First, the small surveillance cohort sample size may limit result generalizability, requiring validation in large multicenter prospective studies. Second, the role of EVX2 methylation in CC recurrence or prognosis remains unclear and needs further investigation. Third, its efficacy in glandular lesions requires verification with larger samples. In addition, tissue heterogeneity between cervical tumor tissues and blood-derived controls may confound the observed methylation signals, supporting cautious interpretation and future validation using normal cervical tissues.

## Conclusions

In summary, we have demonstrated that EVX2 methylation effectively discriminates between normal squamous epithelium and HSIL or CC, thereby offering a non-invasive method to stratify and manage cancer risk in individuals infected with high-risk HPV. Our MALDI-TOF-MS-based assay significantly improved sensitivity compared to conventional cytology and HPV16/18 testing, particularly for detecting HSIL and more severe lesions. Additionally, the diagnostic potential of EVX2 methylation for cervical adenocarcinoma is under further validation.

## Supplementary Information

Supplementary Material 1: Figure. S1. No significant differences in methylation levels at these loci were detected between HPV16/18 infections and other high-risk HPV infections.

Supplementary Material 2: Table S1. Statistical expression of EVX2 in normal mucosa, HSIL, and CC tissues. Table S2. Relationship between EVX2 expression and clinicopathological features in CC. Table S3. Confusion matrices built from EVX2 methylation model in training (A) , validation (B) and additional validation (C) cohorts.

## Data Availability

All the data are included in the article and Supplementary Information files, or available from the authors upon reasonable request. Please contact Wei Wang at ricewang79@126.com for data requests.

## Abbreviations

Hr-HPV: High-risk human papillomavirus
CC: Cervical cancer
IHC: Immunohistochemical
SIL: Squamous intraepithelial lesion
DNMTs: DNA methyltransferases
DMLs: Differential methylation loci
SCC: Squamous cell carcinoma
GEO: Gene Expression Omnibus
TCGA: The Cancer Genome Atlas
MCBs: Methylation-correlated blocks
LASSO: Least absolute shrinkage and selection operator
FFPE: Formalin fixed paraffin embedded
LCT: Liquid-based cytology
ECA: Endocervical adenocarcinoma
HSIL: High-grade squamous intraepithelial lesion
LSIL: Low-grade squamous intraepithelial lesion
MALDI-TOF: Matrix-assisted laser desorption ionization time-of-flight
SAP: Shrimp alkaline phosphatase
ROC: Receiver operating characteristic
MiRNA: MicroRNA
ClN: Cervical intraepithelial neoplasia

## References

[1] Sung H, Ferlay J, Siegel R, L, et al. Global Cancer Statistics 2020: GLOBOCAN Estimates of Incidence and Mortality Worldwide for 36 Cancers in 185 Countries. Cancer J Clin. 2021;71(3):209–49. 10.3322/caac.21660 33538338

[2] Koliopoulos G, Arbyn M, Martin-Hirsch P, et al. Diagnostic accuracy of human papillomavirus testing in primary cervical screening: A systematic review and meta-analysis of non-randomized studies. Gynecol Oncol. 2007;104(1):232–46. 17084886 10.1016/j.ygyno.2006.08.053

[3] Saslow D, Lawson H W Solomond, et al. American Cancer Society, American Society for Colposcopy and Cervical Pathology, and American Society for Clinical Pathology Screening Guidelines for the Prevention and Early Detection of Cervical Cancer. Am J Clin Pathol. 2012;137(4):516–42. 22431528 10.1309/AJCPTGD94EVRSJCG

[4] Fontham E T H, Wolf A M D, Church T R, et al. Cervical cancer screening for individuals at average risk: 2020 guideline update from the American Cancer Society. Cancer J Clin. 2020;70(5):321–46. 10.3322/caac.21628 32729638

[5] Massad L S, Einstein M H, Huh W K, et al. 2012 updated consensus guidelines for the management of abnormal cervical cancer screening tests and cancer precursors. J Lower Genit Tract Dis. 2013;17(5 Suppl 1):S1–27. 10.1097/LGT.0b013e318287d329 23519301

[6] Baylin S B, Jones PA. Epigenetic Determinants of Cancer. Cold Spring Harb Perspect Biol. 2016;8(9):a019505. 27194046 10.1101/cshperspect.a019505 PMC5008069

[7] Han Y, JI L, Guan Y, et al. An epigenomic landscape of cervical intraepithelial neoplasia and cervical cancer using single-base resolution methylome and hydroxymethylome. Clin Translational Med. 2021;11(7):e498. 10.1002/ctm2.498 PMC8288011 34323415

[8] Dovnik A. The Role of Methylation of Host and/or Human Papillomavirus (HPV) DNA in Management of Cervical Intraepithelial Neoplasia Grade 2 (CIN2) Lesions. Int J Mol Sci. 2023;24(7):6479. 37047452 10.3390/ijms24076479 PMC10095339

[9] Yadav Kadianlk, Nanda R. High-risk HPV infection modulates the promoter hypermethylation of APC, SFRP1, and PTEN in cervical cancer patients of North India. Mol Biol Rep. 2020;47(12):9725–32. 33230782 10.1007/s11033-020-05960-z

[10] Huang M, Wang T, Li M, et al. Evaluating PAX1 methylation for cervical cancer screening triage in non-16/18 hrHPV-positive women. BMC Cancer. 2024;24(1):913. 39080593 10.1186/s12885-024-12696-7 PMC11287924

[11] Schmitz M, Wunsch K, Hoyer H, et al. Performance of a methylation specific real-time PCR assay as a triage test for HPV-positive women. Clin Epigenetics. 2017;9:118. 29090037 10.1186/s13148-017-0419-2 PMC5655856

[12] Lin C, Zhu C, Xie M, et al. Analysis of the triage value of multigene methylation testing for CIN2 + in hrHPV-positive patients. Infect Agents Cancer. 2025;20(1):22. 10.1186/s13027-025-00652-4 PMC11974008 40197298

[13] Tian X, Chen D, Zhang R, et al. Quantitative survey of multiple CpGs from 5 genes identifies CpG methylation panel discriminating between high- and low-grade cervical intraepithelial neoplasia. Clin Epigenetics. 2015;7(1):4. 25699113 10.1186/s13148-014-0037-1 PMC4334603

[14] Russell-Goldman E, Hornick JL, Qian X, et al. NKX2.2 immunohistochemistry in the distinction of Ewing sarcoma from cytomorphologic mimics: Diagnostic utility and pitfalls. Cancer Cytopathol. 2018;126(11):942–9. 30376220 10.1002/cncy.22056

[15] Lei S, Li L, Yang X, et al. The association between RAPSN methylation in peripheral blood and breast cancer in the Chinese population. J Hum Genet. 2021;66(11):1069–78. 33958711 10.1038/s10038-021-00933-x

[16] Muthamilselvan S, Raghavendran A. Stage-differentiated ensemble modeling of DNA methylation landscapes uncovers salient biomarkers and prognostic signatures in colorectal cancer progression. PLoS ONE. 2022;17(2):e0249151. 35202405 10.1371/journal.pone.0249151 PMC8870460

[17] Xi T, Zhang G. Epigenetic regulation on the gene expression signature in esophagus adenocarcinoma. Pathol - Res Pract. 2017;213(2):83–8. 28049580 10.1016/j.prp.2016.12.007

[18] Rauch TA, Wang Z, Wu X, et al. DNA methylation biomarkers for lung cancer. Tumor Biology. 2012;33(2):287–96. 22143938 10.1007/s13277-011-0282-2

[19] Ji M, Guan H, Gao C, et al. Highly frequent promoter methylation and PIK3CA amplification in non-small cell lung cancer (NSCLC). BMC Cancer. 2011;11:147. 21507233 10.1186/1471-2407-11-147 PMC3098185

[20] Hua T, Kang S, Li X, et al. DNA methylome profiling identifies novel methylated genes in epithelial ovarian cancer patients with platinum resistance. J Obstet Gynecol Res. 2021;47(3):1031–9. 33403724 10.1111/jog.14634

[21] Arbyn M, Weiderpass E. Estimates of incidence and mortality of cervical cancer in 2018: a worldwide analysis. Lancet Global Health. 2020;8(2):e191–203. 31812369 10.1016/S2214-109X(19)30482-6 PMC7025157

[22] Richardson, L A, El-zein M, Ramanakumar A V, et al. HPV DNA testing with cytology triage in cervical cancer screening: Influence of revealing HPV infection status. Cancer Cytopathol. 2015;123(12):745–54. 26230283 10.1002/cncy.21596

[23] Liang W, Chen Z, Li C, et al. Accurate diagnosis of pulmonary nodules using a noninvasive DNA methylation test. J Clin Investig. 2021;131(10):e145973. 33793424 10.1172/JCI145973 PMC8121527

[24] Mo S, Dai W, Wang H, et al. Early detection and prognosis prediction for colorectal cancer by circulating tumour DNA methylation haplotypes: A multicentre cohort study. EClinicalMedicine. 2023;55:101717. 36386039 10.1016/j.eclinm.2022.101717 PMC9646872

[25] Wang W, Zhu X, Zhang X, et al. Recurrence risk assessment for stage III colorectal cancer based on five methylation biomarkers in plasma cell-free DNA. J Pathol. 2023;259(4):376–87. 36573552 10.1002/path.6047

[26] Fertey J, Hagmann J, Ruscheweyh H, et al. Methylation of CpG 5962 in L1 of the human papillomavirus 16 genome as a potential predictive marker for viral persistence: A prospective large cohort study using cervical swab samples. Cancer Med. 2020;9(3):1058–68. 31856411 10.1002/cam4.2771 PMC6997067

[27] Dong L, Zhang L, HU S Y, et al. Risk stratification of HPV 16 DNA methylation combined with E6 oncoprotein in cervical cancer screening: a 10-year prospective cohort study. Clin Epigenetics. 2020;12(1):62. 32381054 10.1186/s13148-020-00853-1 PMC7204324

[28] Zummeren MV, Kremer W W, Leeman A, et al. HPV E4 expression and DNA hypermethylation of CADM1, MAL, and miR124-2 genes in cervical cancer and precursor lesions. Mod Pathol. 2018;31(12):1842–50. 30135508 10.1038/s41379-018-0101-z

[29] Albulescu A, Plesa A, Fudulu A, et al. Epigenetic approaches for cervical neoplasia screening (Review). Experimental Therapeutic Med. 2021;22(6):1481. 10.3892/etm.2021.10916 PMC8576616 34765022

[30] Kremer W W, Steenbergen R. The use of host cell DNA methylation analysis in the detection and management of women with advanced cervical intraepithelial neoplasia: a review. BJOG: Int J Obstet Gynecol. 2021;128(3):504–14. 10.1111/1471-0528.16395 PMC7818489 32619334

[31] Muresu N, Puci MV, Sotgiu G, et al. Diagnostic Accuracy of DNA-Methylation in Detection of Cervical Dysplasia: Findings from a Population-Based Screening Program. Cancers. 2024;16(11):1986. 38893107 10.3390/cancers16111986 PMC11171015

[32] Fan C, Hu J, Luo T, et al. Analysis of the diagnostic performance of PAX1/SOX1 gene methylation in cervical precancerous lesions and its role in triage diagnosis. J Med Virol. 2024;96(5):e29521. 38727013 10.1002/jmv.29521

[33] Fackler M J, Pleas M, Li Y, et al. Discovery and technical validation of high-performance methylated DNA markers for the detection of cervical lesions at risk of malignant progression in low- and middle-income countries. Clin Epigenetics. 2024;16(1):56. 38643219 10.1186/s13148-024-01669-z PMC11032610

[34] Louvanto K, aro K, Nedjai B, et al. Methylation in Predicting Progression of Untreated High-grade Cervical Intraepithelial Neoplasia. Clin Infect Diseases: Official Publication Infect Dis Soc Am. 2020;70(12):2582–90. 10.1093/cid/ciz677 PMC7286376 31344234

[35] Cheng X, Chai R, Zhang T, et al. A novel methylation-detection panel for HPV associated high-grade squamous intraepithelial lesion and cervical cancer screening. Sci Rep. 2024;14(1):25556. 39462105 10.1038/s41598-024-75047-3 PMC11513036

[36] Gu Y, Chu C, Yuan B, et al. Expression and hypermethylation of JAM and EPB41L3 in cervical squamous cell carcinoma: Clinical significance and applications. Histol Histopathol. 2024;39(8):1043–51. 38213260 10.14670/HH-18-697

[37] Zeng Z, Xie D. Genome-wide identification of CpG island methylator phenotype related gene signature as a novel prognostic biomarker of gastric cancer. PeerJ. 2020;8:e9624. 32821544 10.7717/peerj.9624 PMC7396145

