# Supplementary material for: EVX2 methylation assay enables risk assessment of cervical cancer in high-risk HPV-infected individuals
Source: BMC Cancer. 2026 Jun 8;26:971. doi: 10.1186/s12885-026-16256-z (PMC13471365; doi:10.1186/s12885-026-16256-z)
Supplement: Supplementary file 1 [file 12885_2026_16256_MOESM1_ESM.docx]

**Supplementary Materials for**

**EVX2 Methylation Assay Enables Risk Assessment of Cervical Cancer in High-Risk HPV-Infected Individuals**

**Jingyi, et al.**

The newly designed EVX2 primers were as follows:

F：aggaagagagTTGTTTGTTTTGAAGTTGTTTTTAT

R：cagtaatacgactcactatagggagaaggctAACTAATCAAAATAACCCATACATCCTT

Figures S1. no significant differences in methylation levels at these loci were detected between HPV16/18 infections and other high-risk HPV infections
