# Supplementary material for: EVX2 methylation assay enables risk assessment of cervical cancer in high-risk HPV-infected individuals
Source: BMC Cancer. 2026 Jun 8;26:971. doi: 10.1186/s12885-026-16256-z (PMC13471365; doi:10.1186/s12885-026-16256-z)
Supplement: Supplementary file 2 [file 12885_2026_16256_MOESM2_ESM.docx]

**Supplementary Materials for**

**EVX2 Methylation Assay Enables Risk Assessment of Cervical Cancer in High-Risk HPV-Infected Individuals**

Wu et al.

**Table S1 Statistical expression of EVX2 in normal mucosa, HSIL, and CC tissues**

| **Group** |  |  | **EVX2** |  | **Total** | **P** |
| --- | --- | --- | --- | --- | --- | --- |
|  | - | + | ++ | +++ |  |  |
| Normal Mucosa | 3 | 16 | 38 | 1 | 58 |  |
| HSIL | 12 | 16 | 1 | 1 | 30 | ＜0.001 |
| Normal Mucosa | 3 | 16 | 38 | 1 | 58 |  |
| Cervical cancer | 36 | 17 | 2 | 0 | 55 | ＜0.001 |
| HSIL | 12 | 16 | 1 | 1 | 30 |  |
| Cervical cancer | 36 | 17 | 2 | 0 | 55 | ＜0.01 |
| Stage Ⅰ | 19 | 7 | 1 | 0 | 27 |  |
| Stage Ⅱ | 12 | 6 | 1 | 0 | 19 |  |
| Stage Ⅲ | 3 | 4 | 0 | 0 | 7 |  |

**Table S2 Relationship between EVX2 expression and clinicopathological features in CC**

| **Features** | **Total** | **Negative** | **Positive** | **P** | **λ^2^** |
| --- | --- | --- | --- | --- | --- |
| EVX2 | 55 | 36 | 19 |  |  |
| Age |  |  |  | 0.549 | 0.028 |
| <50 | 24 | 16(66.7%) | 8(33.3%) |  |  |
| ≥50 | 31 | 20(64.5%) | 11(35.5%) |  |  |
| Differentiation |  |  |  | 0.282 | 1.803 |
| Well | 2 | 1(50%) | 1(50%) |  |  |
| Moderate | 14 | 11(78.6%) | 3(21.4%) |  |  |
| Poor | 35 | 22(62.9%) | 13(37.1%) |  |  |
| Micro-invasion | 4 | 2(50%) | 2(50%) |  |  |
| Clinical Stages |  |  |  | 0.178 | 1.811 |
| Ⅰ、Ⅱ | 48 | 33(68.8%） | 15（31.3%） |  |  |
| Ⅲ | 7 | 3（65.5%） | 4（34.5%） |  |  |
| Diameter |  |  |  | 0.347 | 0.478 |
| <2 | 17 | 10(58.8%) | 7(41.2%) |  |  |
| ≥2 | 38 | 26(68.4%) | 12(31.6%) |  |  |
| Invasion depth |  |  |  | 0.273 | 0.806 |
| <1/2 |  | 19(70.4%) | 8(29.6%) |  |  |
| ≥1/2 |  | 14(58.3%) | 10(41.7%) |  |  |
| Lymphnode metastasis |  |  |  | 0.234 | 1.178 |
| Negative |  | 28(68.3%) | 13(31.7%) |  |  |
| Positive |  | 5(50%) | 5(50%) |  |  |
| HPV16/18 |  |  |  | 0.393 | 0.311 |
| Positive | 29 | 18(62.1%) | 11(37.9%) |  |  |
| Negative | 26 | 18(69.2%) | 8(30.8%) |  |  |

**Table S3 Confusion matrices built from EVX2 methylation model in**

**training (A) , validation (B) and additional validation (C) cohorts.**

A

| **Training cohort** | **SIL+CC** | **Normal** |
| --- | --- | --- |
| **SIL+CC** | 32 | 2 |
| **Normal** | 1 | 37 |
| **Sensitivity（%）** | 96.97 |  |
| **Specificity (%)** |  | 94.87 |

B

| **Validation cohort** | **SIL+CC** | **Normal** |
| --- | --- | --- |
| **SIL+CC** | 14 | 3 |
| **Normal** | 1 | 14 |
| **Sensitivity (%)** | 93.33 |  |
| **Specificity (%)** |  | 82.35 |

C

| **Additional validation** | **SIL+CC** | **Normal** |
| --- | --- | --- |
| **SIL+CC** | 9 | 2 |
| **Normal** | 1 | 10 |
| **Sensitivity (%)** | 90 |  |
| **Specificity (%)** |  | 83.33 |
